# Telenursing during the COVID-19 pandemic in the Czech Republic-representative sociological survey

**DOI:** 10.1016/j.heliyon.2023.e19081

**Published:** 2023-08-11

**Authors:** Sylva Bártlová, Ivana Chloubová, Valérie Tóthová, Věra Hellerová, Jana Kimmerová, František Dolák, Olga Shivairová, David Kimmer, Aleš Chrdle

**Affiliations:** a University of South Bohemia in České Budějovice, Faculty of Health and Social Sciences, Institute of Nursing, Midwifery and Emergency Care, J. Boreckého 1167/27, 370 11, České Budějovice, Czech Republic; b University of South Bohemia in České Budějovice, Faculty of Health and Social Sciences, Institute of Humanities in Helping Professions, J. Boreckého 1167/27, 370 11, České Budějovice, Czech Republic; c University of South Bohemia in České Budějovice, Faculty of Health and Social Sciences, Institute of Laboratory Diagnostics and Public Health, J. Boreckého 1167/27, 370 11, České Budějovice, Czech Republic; d Hospital České Budějovice, Infectious Disease Department, a. S., B. Němcové 585/54, 37001, České Budějovice, Czech Republic

## 1 Introduction

The COVID pandemic has brought significant changes in how health care is provided. In a very short period, telemedicine has taken on a prominent role in healthcare [1]. Throughout the pandemic, healthcare professionals have been key players in hospital and community care [2]. Almost overnight, they were expected to learn to use various telemedicine technologies with little or no training. Although the pandemic is subsiding, telemedicine will remain part of the care delivery system and need further development. Therefore, it is necessary to pay attention to best practices in this area and the preparation of health professionals to acquire the competencies required to use it [1].

From the point of view of telecommunications in healthcare, we often encounter overlapping terms telehealth, telemedicine, and eHealth. From the terminological point of view, eHealth can be perceived as health care that may or may not be provided remotely, telehealth as preventive, supportive, and curative health care provided remotely, and telemedicine as medical care that uses telecommunication technologies. Telecare and mhealth (mobile health) represent similar concepts [3]. The American Telemedicine Association (ATA) characterizes telemedicine as a significant and growing component of healthcare. It points to the vital role of nurses who participate in virtually provided care in close cooperation with physicians [4]. Within modern nursing, telenursing is one of the important technological events of the late twentieth century.

Telenursing was a new opportunity during the COVID-19 pandemic. It emphasized education and self-care, helped overcome issues such as restrictions on hospital beds and nursing staff and reduced the cost of treatment, and transitioned into the chronic stage of care [5]. Patient education is essential since it allows nurses to fulfill one of their critical roles, i.e., controlling diseases and related complications [1,6]. Nurses already use various technologies, such as videoconferencing and remote monitoring of basic health indicators (e.g., data on blood pressure or blood glucose levels) [1].

Telenursing aims to improve the quality of care, patient safety, and rapid access to nursing care by overcoming geographical barriers [7].

As noted by the authors [8], telenursing involves using phones, cell phones, SMS, and other communication technologies. On the one hand, there is ever-increasing technological growth; on the other hand, there is a need to increase the availability of nursing services using telenursing in patient care.

Rutledge and Gustin [1] point out that given the increasing need for telenursing, it is also necessary to incorporate this topic into nursing curriculums to prepare nurses to provide this type of care after graduation. The authors mention, for example, that nurses can contribute to the development of telehealth in program management and act as telehealth coordinators. Additionally, nurses can assess the care provided (including incorporating this type of care into nursing standards and guidelines) and have roles as educators and health coaches. Nurses have roles across the entire healthcare system, i.e., in outpatient and hospital spheres.

Based on a pilot study by the authors [9], their varied experience with telenursing in selected EU countries (Spain, Portugal, Poland), they analyze telenursing from a patient perspective or the perspective of a specific nursing specialty, staff management, or promotion. However, what was lacking was a comprehensive practical nursing assessment of the use of telenursing that includes practicing nurses with and without virtual care experience.

According to the World Health Organization [10], discussing the pandemic's end is impossible since individual countries still struggle with its consequences. With this in mind, WHO [10] stresses developing strategies to prevent pandemics and develop plans to minimize their impact. At the same time, it calls for projects to ensure clear and transparent dissemination of information on pandemic diseases. Chao et al. [11] emphasize that nurses need adequate support from their colleagues, supervisors, policymakers, local authorities, and the community to properly prepare for and manage pandemic situations.

Telemedicine technology is a twenty-first-century patient-centered approach that protects patients and health professionals [12]. The development of these technologies is in line with the Regional digital health action plan for the WHO European Region 2023–2030 [13].

The threat of COVID-19 in Europe has necessitated the need for telehealth platforms readily available to patients [5]. In Europe, telemedicine is considered a health service (Directive 2011/24/EU) and an information service (Directive 95/46/EU, Directive 2000/31/EC, and Directive 2002/58/EC). Due to the lack of pan-European uniform medical liability and medical, legislative regulations, a Europe-wide framework still needs to be implemented [14].

For this reason, we were interested in whether nurses in the Czech Republic working in primary care were interested in telenursing and online counseling as a virtual workplace in general practitioner outpatient clinics during the COVID-19 pandemic.

## 2 Research methodology

A descriptive and cross-sectional design was used for this study. A representative sociological survey was conducted. A non-standardized questionnaire was used to collect data, the main objective of which was to map the views, knowledge, and experience of nurses gained during the COVID-19 pandemic and to identify essential issues that must be addressed before the next pandemic.

The sample consists of 1197 nurses. The respondents were selected by quota sampling. The parameters of the nurse sample were constructed based on data from the Institute of Health Information and Statistics at the Ministry of Health of the Czech Republic, valid as of August 19, 2021. The sample size corresponds to the 95% confidence level, and the error interval (Margin error – Confidence interval) was 3% (according to Raosoft).

The sample of nurses included nurses working in both outpatient and inpatient care. The parameters of this sample were derived from nurse demographics in the Czech Republic. The sample of nurses was constructed to correspond to the composition of the nurse population in terms of age, gender, and region (region). These features were intended to be representative of nurses working in the Czech Republic.

Regarding gender, the group consisted of 40 (3.3%) men and 1157 (96.7%) women. The question of gender was formulated with sensitivity to gender and the unique cultural aspects of the Czech Republic. Given that citizens of the Czech Republic often refuse to answer variants other than male/female, we did not include other variants in the questionnaire to avoid reducing the return rate of the questionnaires.

Regarding the age composition of nurses, the 45–54 age group was the most represented, and the 65 and over age group was the least represented.

As part of the research, nurses from all regions of the Czech Republic were approached, and their representation corresponds to the distribution of nurses in the Czech Republic. The deviation from the general population of nurses does not exceed 0.3%; therefore, relative to individual regions, the research sample was representative of nurses in the Czech Republic.

Other socio-demographic and professional features monitored in our research were not meant to be representative since this data is not kept by the information systems in the Czech Republic and could not be assessed for representativeness.

The questionnaire contained a total of 57 questions. The questionnaire is the result of the operationalization of objectives and hypotheses. These were defined based on a theoretical-empirical analysis of the problem under study. An important starting point was the interviews conducted in the qualitative part of the research with nurses and doctors in outpatient and inpatient care, focused on the impact of the COVID-19 pandemic on their work and life. These interviews were used to identify the key areas to be targeted in quantitative research and the key issues that need to be addressed. Based on their operationalization, the first version of the questionnaire was constructed. Its validity was verified using the assessment of experts and target research participants (face and content validity). Content and construct validity was also confirmed through a pre-survey performed using a sample of 128 respondents, in which not only the wording and clarity of the questions were assessed, but also the coverage of areas of nurses’ work and life that might have been affected by the COVID-19 pandemic and their relevance. Construct validity was also tested by a concurrent test of the data obtained from the pre-survey, aimed at identifying the expected correlations between the selected variables.

The issue of nurses' attitudes towards telenursing was saturated with 14 items in the survey. Principal Component Analysis was performed in constructing them, and the Total Variance Explained was calculated. The result of a Rotated Component Matrix allowed us to state that the scale comprises two principal components, which can be considered satisfactory given the number of items.

The questionnaire consists of a section containing sociodemographic characteristics and a section relating to the impact of the pandemic on the work and life of nurses in outpatient and inpatient care. This section is covered by separate closed, semi-open, and open-ended questions; key areas are covered by comprehensive batteries of closed-ended projective questions in statements addressing each topic with response options in the form of a standard four-point scale expressing the level of agreement with the information. A specific area of the research was devoted to questions about the possibility of using an online system for communication with patients. Nurses were asked whether they used an online system to contact patients, what they saw as its positives and negatives, what communication within the online system looked like, and whether they would welcome the creation of virtual educational material in an online form to assist them in dealing with a patient's current medical conditions. Regarding the issue of online communication, nurses were first asked whether they currently use any online system (via the Internet) to communicate with their patients.

The individual battery of questions was analyzed by calculating the mean values for each sub-question in the battery and then comparing them, as well as by calculating the total scores for each battery and then analyzing their possible relationships with the selected characters in the questionnaire.

Reliability measurements for each battery of questions were made using Cronbach's alpha test. Based on its results, it can be concluded that the internal consistency values for each battery of questions ranged from 0.731 to 0.873, indicating a high level of internal consistency.

Our field survey was conducted using standardized guided face-to-face interviews with the respondents. The field survey was conducted throughout the Czech Republic from September 16, 2022, to October 5, 2022. Data collection was provided by 210 professional interviewers from INRES-SONES, v. o.s.

The study was approved by the Ethics Committee of the Faculty of Health and Social Sciences of the University of South Bohemia in Ceske Budejovice under No. 004/2020 on June 15, 2020. The research was anonymous; participation was voluntary, and the survey contained no controversial ethical issues.

Statistical data processing was performed using SASD 1.5.8 (Statistical Analysis of Social Data) and SPSS 28.0. The first level of classification and contingency tables of selected indicators of the second level of classification were prepared. The degree of dependence of the selected characteristics was determined using distribution normality. Based on this analysis, the data was interpreted, and the relevant tables were prepared. Kolmogorov-Smirnov and Shapiro-Wilk tests of normality were performed. Based on their results, additional tests were selected to analyze the data further. According to the distribution and the nature of the variables, the Mann-Whitney *U* test, Spearmen's correlation coefficient, Kruskal Wallis Test, and Pearson Chi-Square were used; control analyses were performed based on ANOVA, Cohen's d was used to measure substantive significance (effect size).

## 3 Results

Currently, only 4.9% of nurses use the online system (via the Internet) to communicate with patients (see Table 1). The remaining 95.1% of nurses said they do not use any online system (Table 2).

**Table 1:** Composition of the sample of nurses by gender, age, region, education, type of medical facility, and years of experience working in health care.

Sex	Absolute	%
Male	40	3.3
Female	1157	96.7
**Age**	Absolute	%
Up to 34	248	21.6
35–44	291	24.3
45–54	367	30.7
55–64	222	18.5
65 and more	59	4.9
**Regions**	Absolute	%
Prague	215	18.0
Central Bohemia	108	9.0
South Bohemian	66	5.5
Pilsen	62	5.2
Carlsbad	30	2.5
Ustecky	72	6.0
Liberec	42	3.5
Hradec Kralovecky	66	5.5
Pardubice	53	4.4
Highland	60	5.0
South Moravia	141	11.8
Olomouc	80	6.7
Zlinsky	63	5.3
Moravian-Silesian	139	11.6
**Education** medical facility	Absolute	%
Intermediate medical	462	38.6
Tertiary professional	315	26.3
Bachelor's education	277	23.1
Master's education	130	10.9
other	13	1.1
**Type of medical facility**	Absolute	%
Primary outpatient care	235	19.6
Specialized outpatient care	149	12.5
Inpatient care	813	67.9
**Length of experience in health care**	Absolute	%
Up to 5 years	218	18.2
6–10 years	280	23.4
11 and more years	699	58.4

**Table 2:** Use of online systems for communication with patients (N = 1197).

Nurses that used an online system to communicate with the patient.	Absolute	%
Yes	59	4.9
No	1138	95.1

The analysis of the relationship between individual sociodemographic characteristics and the use of the online patient communication system showed some statistically significant associations, which are presented in Table 3.

**Table 3:** Relationship between the use of online patient communication systems and the socio-demographic characteristics of nurses in our sample.

The use of online communication with patients	*Value X*^*2*^	*df*	*p*
Sex	0.001	1	0.988
Age	10.479	4	<0.05
Education	3.950	4	0.413
Length of experience in health care	3.192	2	0.203
Type of Medical Facility	30.954	2	<0.001
Place of work during the COVID-19 epidemic	10.422	2	<0.01

*X*^*2*^*- chi-square; df - degrees of freedom*; *p - independence test*.

A statistically significant association was identified between the use of online patient communication and the age of nurses. The use of online patient communication systems (OLPCS) decreased with the increasing age of nurses.

There was a strong link between using OLPCS and the type of medical facility where the nurse works. To a significantly greater extent, nurses working in specialized outpatient care had significantly less experience with OLPCS than nurses in inpatient care. In terms of the place of work during the COVID-19 pandemic, nurses who worked in primary care during this period used OLPCS to a significantly greater extent.

The use of OLPCS was significantly affected by age, type of medical facility, and place of work of a nurse during the COVID-19 epidemic. Statistically significant associations with the other endpoints were not identified.

Nurses who reported using OLPCS (N = 59) mostly communicated via email, SMS, and e-prescriptions. Other systems were rarely mentioned.

Respondents who stated that they did not use OLPCS (N = 1138) were also asked whether they would welcome an opportunity to use it. This question was answered positively by 51.6% of respondents and negatively by 48.6% of nurses.

The analysis of the relationship between individual sociodemographic characteristics and interest in OLPCS among respondents who did not use it revealed some statistically significant relationships, presented in Table 4.

**Table 4:** Relationship between an interest in using online systems and the socio-demographic characteristics of the nurses in our sample.

Interest in using online communication with patients	*Value X*^*2*^	*df*	*p*
Sex	0.039	1	0.846
Age	3.583	4	0.465
Education	15.010	4	<0.01
Number of years of healthcare experience	15.937	2	<0.001
Type of Medical Facility	10.766	2	<0.01
Place of work during the COVID-19 epidemic	6.156	2	<0.05

X^2^ - chi-square; df - degrees of freedom; p - independence test.

A statistically significant association was identified between an interest in using OLPCS and the education levels of those who have yet to use the system. Respondents with higher levels of education showed significantly greater interest in using OLPCS. We also noted that respondents with less experience in health care (6–10 years) showed significantly greater interest in OLPCS; this interest was significantly less among nurses with more experience (>11 years).

The type of medical facility also significantly influences interest in using OLPCS. The appeal was significantly greater in nurses working in primary outpatient care than in-patient care. It was also found that nurses who worked in primary care during the pandemic were significantly more interested in introducing an online system to communicate with patients.

Interest in using OLPCS among those nurses who do not yet use this system was significantly influenced by education, length of experience in healthcare, type of medical facility, type of hospital, and the nurses’ workplace during the COVID-19 epidemic. Statistically significant associations with other endpoints were not identified.

When asked what nurses would expect from an online system, the nurses stated that they would especially like the online system to serve in communication with patients, primarily to help schedule regular preventive check-ups. Almost 2/3 (65.6%) of nurses identified this function. Nurses also wanted the online system to order extra examinations (53.3%), to provide administration and confirmations (49.5%), to keep track of patient medications (48.2%) (i.e., what medications the patient is taking and when a refill prescription will be needed), and for an overview of the patient's health status (41.3%).

Fewer nurses wanted the online system for patient education conducted by nurses (35.3%), for health checks with the potential to schedule an online consultation (i.e., a call with a doctor via the Internet (32.2%)), and for health checks with the possibility to schedule online consultations with a nurse via the Internet (31.4%).

## 4 Discussion

The research aimed to determine whether nurses in the Czech Republic, who worked in primary care, reported using or being interested in using telenursing and online counseling as a virtual workplace in GP surgeries during the COVID-19 pandemic. Currently, only 4.9% of nurses in the Czech Republic use an online system (via the Internet) to communicate with patients. The remaining 95.1% of nurses reported that they did not use any such methods. The interest in using an online system for communication with patients among those nurses who do not use online systems was significantly influenced by education, length of experience in healthcare, where they worked (i.e., the type of healthcare facility), type of hospital, and where the nurse worked during the COVID-19 pandemic.

Given the need for preparedness for possible pandemics in the future, we need to consider telemedicine as a new way to organize patient care. It is interesting to note that nurses in the Czech Republic expect online systems to be used to communicate with patients regarding arranging regular preventive check-ups, making appointments for emergency check-ups, providing administration and necessary certificates, keeping track of patient medications (what medications the patient is taking and when a refill will be needed), and for keeping track of patient health status. Nurses were least interested in using online systems for patient education.

Totten et al. [3], based on a review of available studies, point to the benefits of telehealth in specific patient groups. In addition to improving ongoing results, the studies demonstrate increased patient satisfaction with the care provided. A significant advantage of telehealth is home monitoring for patients with chronic diseases, communication, and counseling for these patients, and providing psychotherapy as part of mental health care. Currently, only 4.9% of nurses in the Czech Republic use an online system (via the Internet) to communicate with patients. The remaining 95.1% of nurses said they do not use any such system.

Respondents not using online systems (N = 1138) were also asked whether they would welcome the opportunity. This question was answered positively by 51.6% and negatively by 48.6% of nurses. Nurses who reported using an online system (N = 59) did so mostly via e-mail, SMS, and e-prescriptions. Other methods were rarely mentioned. New models of remote healthcare require health professionals to acquire the necessary information and communication technologies (ICT) skills [15]. The use of ICT in the provision of health care allows for more active involvement of non-medical healthcare professionals, which, however, cannot be done in the future without defining the required competencies for remote care relative to individual care.

In their results, Opatrný et al. [16] point out that healthcare facilities in the Czech Republic are well-equipped with the hardware needed to provide telemedicine. However, they point to the continuing use of paper-based documentation and the resistance posed by doctors and patients. Much of the resistance is linked to the increased need for ICT skills, protection of patient data, and awareness of telemedicine opportunities. A relatively significant handicap in this area in the Czech Republic is that a unified information technology infrastructure to process and transmit health records data has not been created. According to the authors, this is somewhat related to the only very recent approval of the telemedicine law.

The results of a systematic review study by Novita et al. [17] showed that using SMS, phone, mobile, and videophone effectively improved treatment outcomes in all studies. Many positive effects of telenursing have been confirmed, such as promoting the quality of care, improving treatment outcomes, reducing treatment costs, reducing the need for visits, involving the patient and the family in care decisions, and the potential for careful monitoring of patients. Kristová et al. [18], based on the analysis of the Telesestra project, state that education, in general, and education through websites can effectively complement traditional nursing care.

Since distance counseling is one of the essential tools of the process, we found it interesting that nurses were not enthusiastic about using OLPCS for education conducted by nurses (35.3%). Chang et al. [19] found that the willingness of nurses to use ICT was influenced by their attitude, support, and encouragement from superiors and their level of ICT knowledge. To some extent, this corresponds with the results of an overview by Firouzkouhi et al. [5], who state that the main problems of telenursing during COVID-19 include difficulties with implementation, insurance coverage, nurse resistance, the problem of continuous care and changing the roles of nurses, infection, and the development of nursing knowledge. Another study showed that COVID-19 patients who were quarantined and suffering from psychological problems caused by loneliness and death anxiety had reduced complications when treated with telenursing [20].

Another reason for implementing telenursing is the geographical distance of healthcare users from health services. Many people infected with COVID-19 live at the edge of towns or villages without easy access to adequate medical care. By removing these barriers, telenursing helps patients better fight COVID-19. In addition, telenursing can help prevent the spread of disease and may also be effective in reducing mortality and helping to identify critically ill patients [21]. With telenursing, patients can be educated without time and space limitations. This, in turn, can reduce patient costs and improve the quality of nursing care. Also, after the patient is discharged from the hospital, continuity of care is maintained, and the recovery period is reduced [22].

Despite the challenges, telenursing has many advantages that have made it effective and efficient during COVID-19 [7]. In countries where telenursing (especially using mobile phones) is well-developed, there is strong and convincing evidence regarding its efficacy and benefits [23]. The coronavirus disease has accelerated the adoption of telemedicine globally. The current consortium critically examines the telemedicine frameworks, identifies gaps in their implementation, and investigates the changes in telemedicine framework/s during COVID-19 globally [24]. Since telenursing is still a relatively new method in the Czech Republic, intensive research is still needed to prove its effectiveness and how it should be used. Future research must evaluate long-term outcomes, nurse and patient satisfaction, and financial sustainability.

Our research aimed to contribute to developing virtual educational material for patients using adult general practitioner outpatient clinics. The material will be based on nurse opinions. We also want to test the potential use of virtual online environments by acute care nurses for education during the discharge to the home environment and as motivation for intensive patient-nurse collaboration in primary care. Subsequently, we intend to validate the virtual patient educational material with nurses working with adult general practitioners.

### 4.1 Strengths and limitations

Limitations of our study include the disadvantage of using a non-standardized questionnaire, as it may sometimes bias respondents’ views. The nurses in our study expressed their attitudes, opinions, and experiences. Only a small percentage of primary care nurses use online (via the internet) to communicate with patients, mainly through emails, SMS, and e-prescriptions.

The strength of the study is its representativeness. Ours is the first representative research on using telenursing in nurses during the COVID-19 pandemic in the Czech Republic.

## 5 Conclusion

Technological advances have changed the role of nurses, especially in areas such as telenursing. Considering nurses’ low use of online systems in communication with patients, we recommend supporting and helping nurses in the Czech Republic resolve this situation. We encourage using creative methods and telenursing to provide remote nursing care. Telenursing will likely be especially important in the event of another pandemic.

Based on the research findings, a virtual online environment/nurse-led consultation will be created to support the management of epidemic/pandemic, chronic and civilization diseases in primary care nurses.

## Author contribution statement

Sylva Bartlova: Ivana Chloubová: Conceived and designed the experiments; Analyzed and interpreted the data; Contributed reagents, materials, analysis tools or data; Wrote the paper. Valérie Tóthová: Conceived and designed the experiments; Performed the experiments; Analyzed and interpreted the data. Věra Hellerová: Conceived and designed the experiments; Performed the experiments; Contributed reagents, materials, analysis tools or data. Jana Kimmerová: František Dolák: Performed the experiments; Analyzed and interpreted the data. Olga Shivairova: David Kimmer: Aleš Chrdle: Conceived and designed the experiments; Performed the experiments.

## Data availability statement

Data associated with this study hs been deposited at Faculty of Health and Social Sciences, Department of Nursing and Midwifery, U Výstaviště 26, 370 04 České Budějovice.

## Funding

Supported by the 10.13039/501100003243Ministry of Health of the Czech Republic, Czech Republic, grant nr. NU21-09-00300. All rights reserved.

## Ethical approval

The research was conducted per ethical principles and was approved by the relevant ethics committee under no. 004/2020 on June 15, 2020.

## Declaration of competing interest

The authors declare that they have no known competing financial interests or personal relationships that could have appeared to influence the work reported in this paper.
